# Infective Endocarditis Presenting as Subarachnoid Hemorrhage: An Appeal for Caution

**DOI:** 10.7759/cureus.1176

**Published:** 2017-04-19

**Authors:** Talal Asif, Amena Mohiuddin, Badar Hasan, Amgad Masoud

**Affiliations:** 1 Department of Internal Medicine, University of Missouri Kansas City (UMKC)

**Keywords:** infective endocarditis, non aneurysmal subarachnoid hemorrhage

## Abstract

Spontaneous subarachnoid hemorrhage (SAH) as the presenting feature of infective endocarditis (IE) is rare. It has classically been described in association with the rupture of intracranial mycotic aneurysms (ICMA). Here we describe a very rare case of non-aneurysmal spontaneous SAH in a patient with IE. The patient originally presented with a headache and low-grade fever. Neuroimaging including computed tomography (CT) and magnetic resonance imaging (MRI) of the head revealed SAH. She was eventually diagnosed with streptococcus gallolyticus (formerly streptococcus bovis) IE. Through this case, we advocate consideration of the diagnosis of IE in patients with SAH so that there is timely recognition of this reversible but serious disease.

## Introduction

Spontaneous subarachnoid hemorrhage (SAH) as the presenting feature of infective endocarditis (IE) is rare. It has only been described in a few case reports with most cases having ruptured mycotic aneurysms as the documented source of hemorrhage [1]. Here we report an interesting case of SAH without any identifiable source of hemorrhage or evidence of aneurysmal rupture. Our patient was eventually diagnosed with streptococcus gallolyticus (formerly streptococcus bovis) IE. Through this case, we advocate consideration of the diagnosis of IE in patients with SAH so that this critical diagnosis can be made in a timely manner to enhance patient safety.

## Case presentation

A 50-year-old female, with a past medical history of essential hypertension and type 2 diabetes mellitus, was admitted from the emergency room (ER) with the chief complaint of generalized weakness and headache for one week.

To give a brief background, she had been admitted at our hospital twice over the past three months with dizziness and syncope. During the first hospitalization three months prior, magnetic resonance imaging (MRI) of the head had shown no acute infarct or hemorrhage. A computed tomographic (CT) angiogram of the head and neck performed at the same time revealed normal intracranial vessels with no stenosis, aneurysms, or arteriovenous malformations (AVM). A transthoracic echocardiogram (TTE) showed no abnormality. The patient’s symptoms improved with supportive care, and she was discharged in stable condition.

The patient was hospitalized again one month later with an episode of syncope. An MRI of the head was repeated that showed a small area of FLAIR hyperintense signal in the left pre-central gyrus that was new from the previous exam. A CT angiogram of the head and neck was also repeated that showed no aneurysms or AVM. All major dural venous sinuses were patent. An electroencephalogram (EEG) revealed no epileptiform activity. The patient underwent extensive infectious and inflammatory workup because of the differentials of infectious versus inflammation on the basis of her MRI findings. Lumbar puncture (LP) and cerebrospinal fluid (CSF) exam, CSF cultures, vasculitic workup, blood cultures, and urine cultures were negative. There was no fever documented at any point during her hospital course. She was discharged with close follow-up and had been faring well on clinic visit two weeks later.

The patient presented to the emergency room (ER) one week later with the complaint of weakness and mild generalized headache. On physical examination, her blood pressure was 135/75, pulse 102 beats/minute, temperature 100.5°F and respiratory rate of 18/minute. Neurological exam was non-focal with no signs of meningeal irritation. She had poor oral hygiene with significant chronic periodontitis and gingivitis. Her cardiovascular exam did not show any abnormal heart sounds. Abdominal, respiratory, and skin exam was unremarkable.

**Figure 1:**
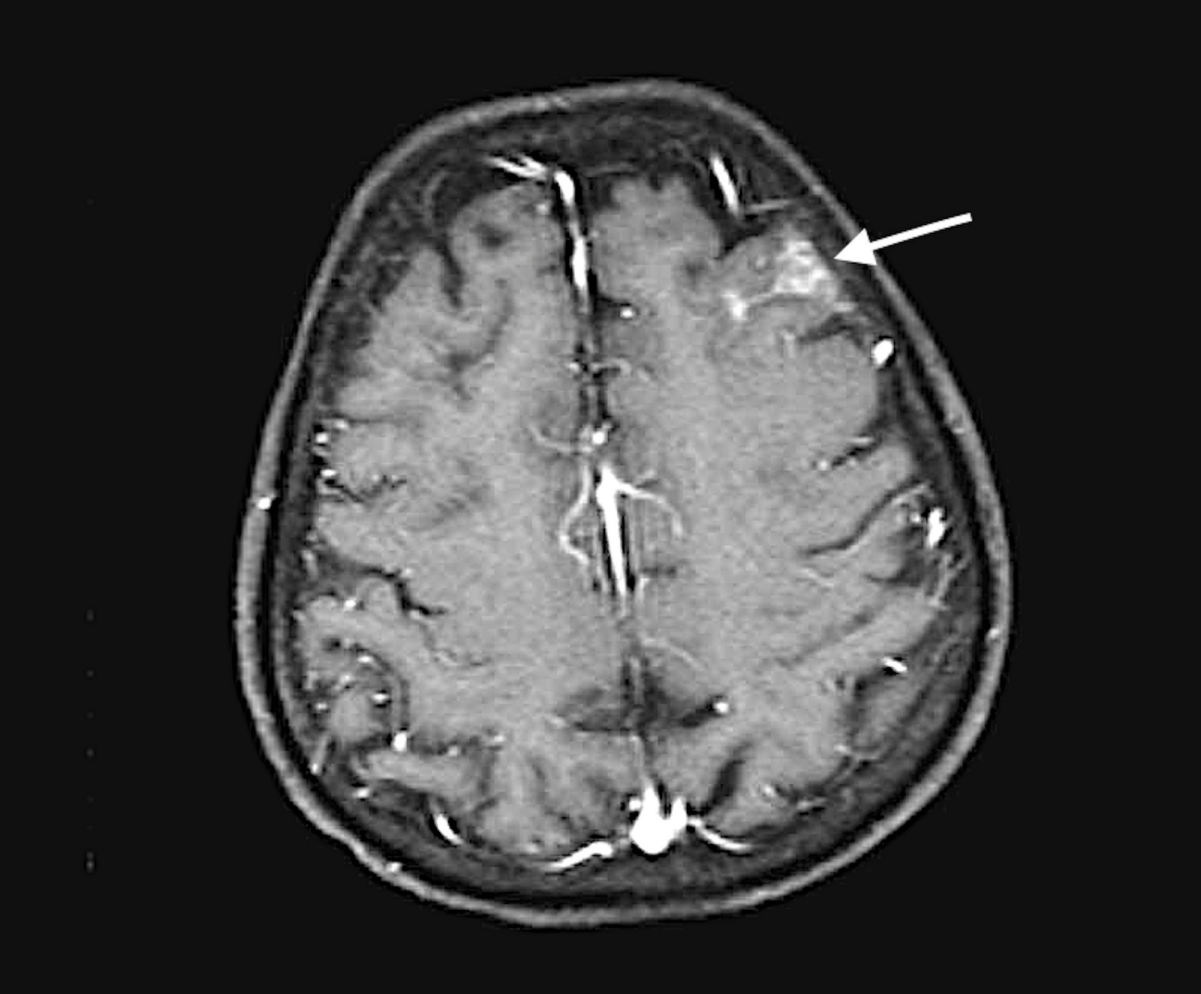
Magnetic resonance imaging (MRI) of brain depicting left frontal lobe subarachnoid hemorrhage (arrow).

Baseline labs especially complete blood count (CBC), basic metabolic panel, and liver panel were within normal limits. Notably, there was no leukocytosis. At this juncture, blood and urine cultures were obtained. CT scan of the head indicated SAH in the left frontal sulci. MRI brain was ordered that revealed new SAH involving multiple cerebral hemispheres, largest one in the left frontal lobe region (Figure 1).

Previously described abnormality in the left pre-central gyrus had resolved. The patient denied any history of trauma. CT angiogram and venogram was performed that showed normal intracranial vessels with no aneurysms and AVM. Dural venous sinuses were again reported to be patent. Magnetic resonance angiogram (MRA) of the head was done subsequently that showed no intracranial vascular abnormality.

**Figure 2:**
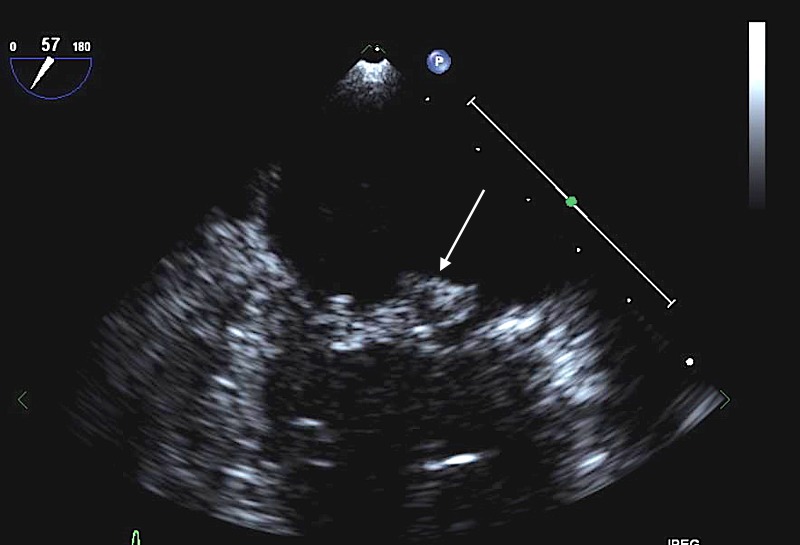
Transesophageal echocardiogram (TEE) showing mitral valve vegetation (arrow).

**Figure 3:**
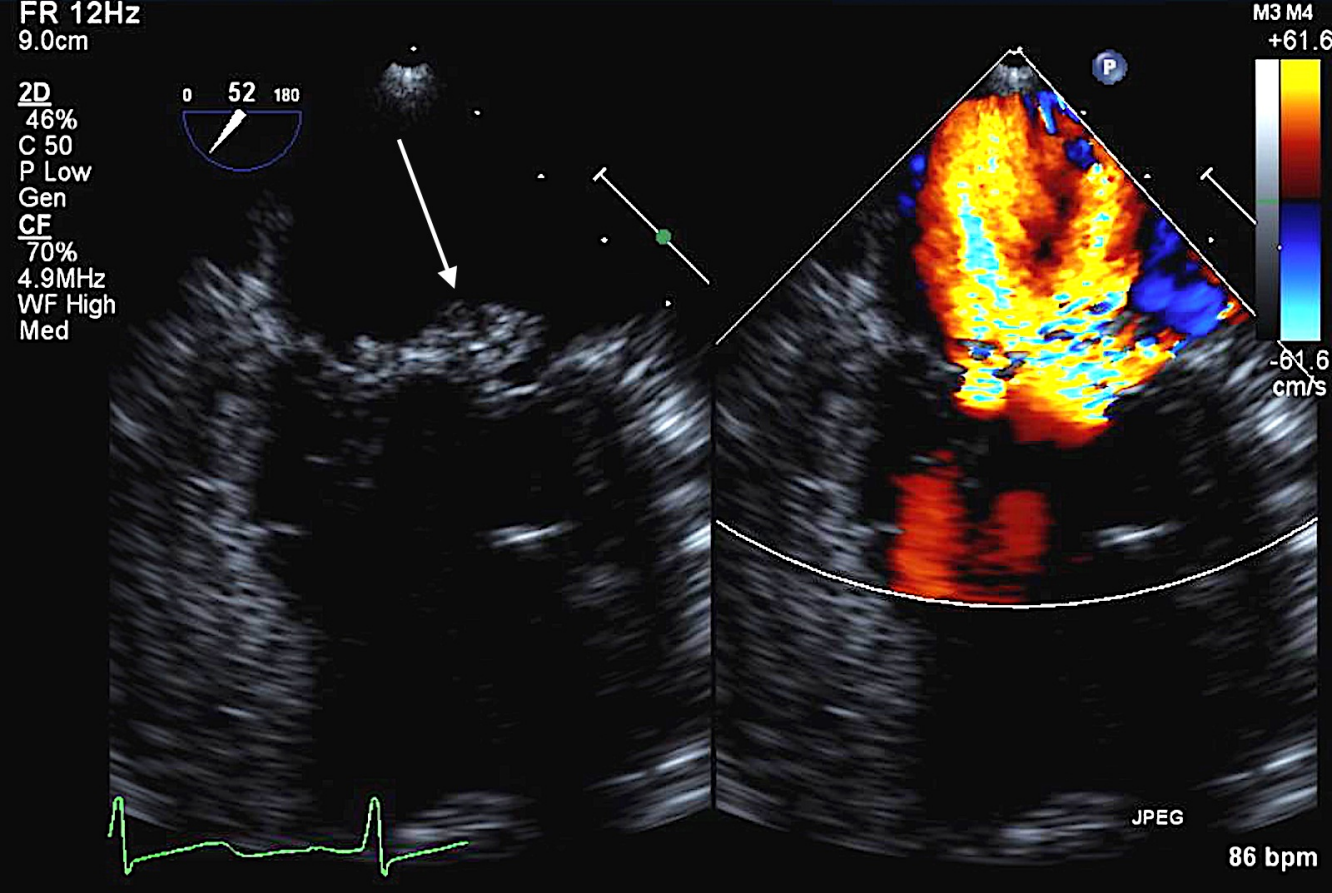
Figure showing mitral regurgitation on the right and the mitral valve vegetation (arrow) on the left.

Neurology consultation was requested. LP was performed with CSF exam unrevealing. Vasculitic workup was repeated which showed an elevated erythrocyte sedimentation rate (ESR) of 102 and C-reactive protein of 3.2. Antinuclear antibody (ANA), rheumatoid factor (RA), and antineutrophil cytoplasmic antibody (ANCA) levels were negative. Complement levels were normal. Blood cultures returned positive for streptococcus gallolyticus (formerly streptococcus bovis). Given the poor oral hygiene, streptococcus gallolyticus bacteremia and SAH, diagnosis of IE was made. Blood cultures were drawn and she was started on penicillin and gentamycin. Blood cultures were positive for streptococcus gallolyticus again. TTE was repeated that showed no vegetations. However, she had new mild mitral regurgitation as compared to TTE done three months previously. Transesophageal echo (TEE) was ordered. The patient first underwent removal of her infected teeth after which TEE was performed which revealed a 0.9 × 0.7 cm vegetation on the posterior mitral leaflet with severe mitral regurgitation (Figures 2-3).

Her ejection fraction was 55-60%. Given the streptococcus bovis species bacteremia, colonoscopy was done which did not show any abnormality.

With treatment, her blood cultures became negative and her symptoms of headache, fever, and weakness resolved. The total duration of treatment was two weeks of intravenous penicillin and gentamicin followed by four weeks of intravenous penicillin monotherapy. SAH had resolved on follow-up MRI head one month later.

## Discussion

Neurologic manifestations of IE are seen in approximately 40% of patients [2]. They have been described as the presenting symptom in nearly 47% of cases [2]. Neurological complications consist of ischemic stroke or transient ischemic attack (TIA), cerebral hemorrhage, cerebral microbleeds, cerebral abscess, mycotic aneurysms, meningitis, or toxic encephalopathy [2]. These have a negative impact on the outcome with a reported 52% mortality at one year in patients with IE complicated by stroke [3].

Cerebral hemorrhage constitutes 12% to 30% of neurological complications in IE [2, 4]. The pathological mechanisms are wide-ranging. Hemorrhagic conversion of infarcted area secondary to septic emboli is the most common mechanism defined [5]. Another process implicated is rupture of intracranial mycotic aneurysms (ICMA) [6]. Asymptomatic cerebral microbleeds are increasingly being described as the most common cerebrovascular lesions [7]. Microbleeds are postulated to develop due to immunologic vasculitis and are an independent risk factor for the development of intracranial hemorrhage [7].

SAH in the setting of IE is uncommon, described only in case reports [1]. It is classically associated with rupture of ICMA [6]. Non-aneurysmal spontaneous SAH as a complication of IE is infrequent and has only been described in a few case reports. Its pathogenesis remains unknown. Some of the hypothesized mechanisms incorporate focal arteritis, vessel rupture, and spontaneous occlusion of leaking aneurysm after hemorrhage [1]. Patients often present with non-specific constitutional symptoms, making this vital diagnosis even more challenging [1]. Headache was also reported by patients as diffuse and vague as opposed to the sudden severe headache classic for aneurysmal SAH [1].

American heart association (AHA) scientific statement on IE recommends computed tomographic angiography (CTA) and magnetic resonance angiography (MRA) as the initial test of choice in patients with neurological symptoms such as headache [8]. CTA and MRA have been shown to have a high sensitivity and specificity (>97%) in the detection of cerebral aneurysms 3 mm or larger in size [9-10]. CTA and MRA have especially high sensitivity and specificity in the detection of ruptured intracranial aneurysms [9-10]. Conventional angiography is deemed reasonable in patients with negative CTA and MRA with a high suspicion of ICMA. Conventional angiography however is associated with neurologic complications in 2.6% of patients [10]. Cerebral arteriography was not pursued in our patient due to low suspicion for occult aneurysmal bleed.

Early institution of appropriate antibiotic therapy constitutes the cornerstone of management of patients with non-aneurysmal SAH and IE [6]. There are no clear guidelines on endovascular or surgical intervention in the absence of aneurysmal dilatation of intracranial vessels. We recommend conservative management in patients with spontaneous SAH in the absence of seizures, encephalopathy, or focal neurological deficits. Repeat follow-up imaging preferentially with MRI is also advised [6].

## Conclusions

Our case report carries immense significance because IE is a reversible and manageable cause of SAH. Timely recognition and treatment of our patient lead to a favorable outcome. There is still much to learn about the pathophysiology, diagnosis, and management of non-aneurysmal SAH in the background of IE. Given the dearth of data in literature, case reports such as these will serve the purpose of providing clinical guidance and direction.
